# Palindromic sequence-targeted (PST) PCR: a rapid and efficient method for high-throughput gene characterization and genome walking

**DOI:** 10.1038/s41598-019-54168-0

**Published:** 2019-11-27

**Authors:** Ruslan Kalendar, Alexandr V. Shustov, Mervi M. Seppänen, Alan H. Schulman, Frederick L. Stoddard

**Affiliations:** 1 Department of Agricultural Sciences, Viikki Plant Science Centre and Helsinki Sustainability Centre, University of Helsinki, P.O. Box 27 (Latokartanonkaari 5), FI-00014 Helsinki, Finland; 2 PrimerDigital Ltd, FIN-00710 Helsinki, Finland; 3 National Center for Biotechnology, Korgalzhin hwy 13/5, 010000 Astana, Kazakhstan; 4 Institute of Biotechnology and Viikki Plant Science Centre, University of Helsinki, P.O. Box 65, FI-00014 Helsinki, Finland; 5 Natural Resources Institute Finland (Luke), Latokartanonkaari 9, FI-00790 Helsinki, Finland

**Keywords:** PCR-based techniques, Comparative genomics

## Abstract

Genome walking (GW) refers to the capture and sequencing of unknown regions in a long DNA molecule that are adjacent to a region with a known sequence. A novel PCR-based method, palindromic sequence-targeted PCR (PST-PCR), was developed. PST-PCR is based on a distinctive design of walking primers and special thermal cycling conditions. The walking primers (PST primers) match palindromic sequences (PST sites) that are randomly distributed in natural DNA. The PST primers have palindromic sequences at their 3′-ends. Upstream of the palindromes there is a degenerate sequence (8–12 nucleotides long); defined adapters are present at the 5′-termini. The thermal cycling profile has a linear amplification phase and an exponential amplification phase differing in annealing temperature. Changing the annealing temperature to switch the amplification phases at a defined cycle controls the balance between sensitivity and specificity. In contrast to traditional genome walking methods, PST-PCR is rapid (two to three hours to produce GW fragments) as it uses only one or two PCR rounds. Using PST-PCR, previously unknown regions (the promoter and intron 1) of the *VRN1* gene of Timothy-grass (*Phleum pratense* L.) were captured for sequencing. In our experience, PST-PCR had higher throughput and greater convenience in comparison to other GW methods.

## Introduction

Genome walking (GW) refers to a collection of methods that capture unknown (unsequenced) genomic regions that are contiguous with and adjacent to a known DNA sequence. Before the advent of next-generation sequencing (NGS), capturing of unknown DNA was based on GW or screening of genomic libraries. The initial GW technology also utilized libraries and screening (e.g. use of a sequenced fragment as a probe for hybridization). PCR-based methods are currently predominant among GW techniques because they are rapid and their use avoids construction of large libraries. The first applications of PCR as an alternative to hybridization were reported in the late 1980s^1^. PCR-based GW can be employed when it is possible to select at least one sequence-specific primer (SSP) that anneals to the DNA of interest. A paired companion to the SSP is a walking primer that is intended to solve the problem of annealing (with the greatest achievable specificity) to an unknown target. In principle, all significant improvements to PCR-based GW technologies have been aimed at controlling the balance between sensitivity (i.e. the ability to generate a GW product) and specificity (avoiding generation of unwanted products).

The best walking primer for a particular application reflects a good guess, or a working hypothesis about the unknown target DNA. Often, minimal *a priori* knowledge about the target, e.g. a protein alignment for a similar target, requires the use of degenerate oligonucleotides as walking primers. In turn, applying the degenerate oligonucleotides in PCR with complex templates (genomic DNA) is often accompanied by nonspecific amplification; this is a driving force behind the development of new methods, such as PST-PCR as described here. A review and the systematics of PCR-based GW methods have been presented^2^. Various PCR-based GW approaches have been used to capture DNA fragments from representatives of all kingdoms of life^2^. More recently, GW-like techniques have proven valuable for studies in which DNA capture itself is not the ultimate goal; such studies include population genetics and diversity studies on populations and species^3–17^.

Current PCR-based GW methods are more elaborate than the protocols initially used, and some patented techniques are available for researchers in the form of commercial GW kits. Two widely published commercial technologies are the GenomeWalker Kits (Clontech & Takara) and the APAgene GW kit (Bio S&T)^2^. With regard to these systems, a comparison of the modern methods for capturing unknown DNA illustrates a diversity of operable approaches. The GenomeWalker Kits are ligation-based, requiring ligation of adaptors to blunt-ended fragments of target DNA; the approach is called “suppression PCR” because the generation of side products is suppressed during PCR.

The GenomeWalker GW process starts with the creation of “libraries” of DNA fragments, of which each library consists of digestion products of starting DNA. Restriction endonucleases yield blunt ends to which synthetic adaptors are subsequently ligated. To increase chances of success, the manufacturer recommends simultaneously performing the GW on four libraries, each produced with a different restriction enzyme. The molecular design of the proprietary adaptor is important, as the design provides for increased specificity. The adaptor is composed of two synthetic oligonucleotides with uneven lengths, so that the adaptor has the protruding 5′-end. The 3′-end of the recessed strand in the adaptor is blocked by amination (3′-NH_2_) to prevent the polymerase from extending this 3′-end during a PCR. The PCR is performed with a user-provided SSP in combination with a universal adaptor primer that targets the adaptor’s protruding end. This primer combination, in theory, allows amplification of only the desired GW products, because the amplified targets must have annealing sites for both the SSP and adaptor primer. The method has been designed to inhibit amplification of unwanted (Random Amplified Polymorphic DNA (RAPD) like) products that have the adaptor primer at both ends. For this purpose, it relies both on the 3′-aminated adaptor described above being present in one strand, and then formation during the PCR annealing step of a “panhandle” structure that diminishes (suppresses) annealing of the adaptor primer on the other strand (hence the name “suppressive PCR”).

The APAgene GW kit uses a three-round nested PCR approach. Each round is performed with a combination of one user-provided SSP and a walking primer from the kit; the kit provides three walking primers with distinctive sequences. A successful capture in the first round depends on annealing of a walking primer to the unknown target DNA. The annealing is achieved by using a walking primer that contains a highly degenerate sequence at the 3′-end. Two more rounds are performed to increase the yields of specific GW products. The second and third rounds are performed with combinations of nested SSPs and so-called tagging primers. The tagging primers are designed to be capable of hairpin formation at annealing temperatures. The following two features of the APAgene process are essential for increasing specificity of the amplification: the first-round PCR starts with a single-strand amplification phase that is a repetitive extension from the outer SSP (this phase is intended to increase the template amount for degenerate primer binding); formation of hairpins in the tagging primers make nonspecific annealing less efficient.

In general, relying on walking primers that have degenerate sequences is common to the majority of GW methods. The use of degenerate primers has the drawback of nonspecific annealing and amplification, which may necessitate a tedious search for the best PCR conditions. One frequent obstacle is that annealing temperatures for the SSPs and degenerate primers are very different; the SSPs are selected to anneal with high specificity and have high melting temperatures (Tm), whereas degenerate primers require a low T_m_^8,15,18^. To achieve efficient amplification from two primers having a different T_m_, an elaborate thermal cycling profile has to be used. Such a profile consists of thermal cycles with different annealing temperatures that follow each other or alternate in a defined fashion.

Solving a similar task (pairing primers of different T_m_) resulted in development of the thermal asymmetric interlaced PCR (TAIL-PCR) method. TAIL-PCR is a GW method that utilizes three consecutive PCR rounds with nested SSPs together with a shorter arbitrary degenerate primer. Each round is performed with one SSP (three different high-T_m_ primers are used in the whole process) and the same short arbitrary degenerate primer with a low Tm. The three rounds differ in thermal cycling profiles. The thermal cycling profile used in the first-round PCR is the most elaborate and represents combinations of high-stringency cycles (annealing temperature 63 °C), reduced-stringency cycles (annealing temperature 44 °C), and low-stringency cycles (annealing temperature 30 °C)^19,20^. For example, the first round may include five high-stringency cycles, followed by one low-stringency cycle, followed by ten reduced-stringency cycles and finishing with twelve “supercycles” (each consisting of two high-stringency cycles and one low-stringency cycle)^20^. The product of the first round is diluted 1/1000 and used as a template in the second-round PCR. The second round includes ten supercycles. Again, the diluted product of the second round is re-amplified in the third round, which resembles a common PCR^20^. Although such elaborate amplification schemes provide the opportunity to optimize experimental conditions, optimization is time consuming and does not guarantee success.

Difficulties associated with the use of degenerate primers in PCR motivated attempts to limit primer degeneracy and to devise walking primers that anneal to genomic DNA not randomly but arbitrarily, at unknown but defined sites. This idea is expressed in its most consistent form in the partially overlapping primer (POP) PCR and stepwise partially overlapping primer (SWPOP) PCR methods^21,22^. In the POP and SWPOP methods, walking primers have completely defined sequences with no degenerate or modified positions. To allow annealing of the walking primers, just one cycle in a thermal cycling profile has extremely low (25 °C) annealing temperature. Sets of walking primers with different sequences were devised for empirical trials for both methods. Both methods require using three nested SSPs. To suppress amplification of by-products that are flanked with walking primers, a three-round PCR is used with different SSPs and walking primers. Sequences of the walking primers and temperature profiles are devised so that annealing of a walking primer to template DNA occurs only once during each round. It was reported that a specific product outcompetes RAPD-like products in the POP and SWPOP processes.

Each GW method has advantages and limitations. For example, despite recently published improvements, TAIL-PCR tends to generate significant amounts of side products. On the other hand, ligation-dependent methods are extremely sensitive to template quality. Manufacturers of ligation-dependent GW kits inform users that the starting DNA must be of much higher quality than generally considered necessary for conventional PCR; they also provide ready-to-use libraries for several species. Ligation-dependent GW methods have slow throughput and are not preferable for population studies or those having available only limited or degraded DNA.

Here we report a novel method that we named palindromic sequence-targeted PCR (PST-PCR). PST-PCR is rapid (requiring only one or two PCR rounds) and demonstrates an excellent combination of sensitivity and specificity. Like the above-described POP and SWPOP methods, the PST-PCR method uses walking primers that anneal to a template not randomly but arbitrarily, although this feature was achieved in a different fashion^21,22^. In our hands, PST-PCR method has a higher efficiency, cost-effectiveness, and sensitivity than previously published methods^4,8,11,17,20–22^.

The central element of PST-PCR technology is a distinctive design of walking primers that enables annealing to short palindromic sequences, or more specifically, to restriction sites (herein referred to as PST sites, not to be confused with restriction enzyme *Pst* I sites). The walking primers, named palindromic sequence-targeted (PST) primers, anneal to the PST sites that have unknown locations in unsequenced DNA. However, the annealing is not random, as the sequence of the PST site itself is defined. Moreover, the annealing of the PST primer occurs even at a high Tm, which is recommended in the PST-PCR protocol. A PST primer has a palindromic sequence of 6 nt at the 3′-end, followed by a degenerate sequence of sufficient length (i.e. 8–12 nt) to allow binding to an unknown target, and completed with an adaptor region of defined sequence at the 5′-end. The principal feature of the PST-PCR protocol is use of high annealing temperatures throughout the PCR amplifications. The high T_m_ prevents annealing to sequences other than PST sites and contributes to specific amplification of a GW product. Only two or even a single PCR round of about 30–36 cycles is needed to produce a PST-PCR product. Accordingly, PST-PCR can be performed in two to three hours. The PST primer is used in combination with an SSP in the first-round PCR. The same SSP or a nested SSP is used with a tail primer during the second-round PCR. The short tail primer anneals to the adaptor sequence at the 5′-end of the PST primer. In addition, PST-PCR is not very sensitive to template quality; PST-PCR requires starting DNA of a quality similar to that suitable for common PCR.

In this study, the power of PST-PCR was demonstrated during GW by capturing presently unknown sequences from the promoter and intron 1 of the *VERNALIZATION 1* (*VRN1*) gene from Timothy-grass (*Phleum pratense* L), as a representative of the *Poaceae* family.

## Results and Discussion

### PST-PCR principles, starting protocol, and possibility of customization

**Figure 1:**
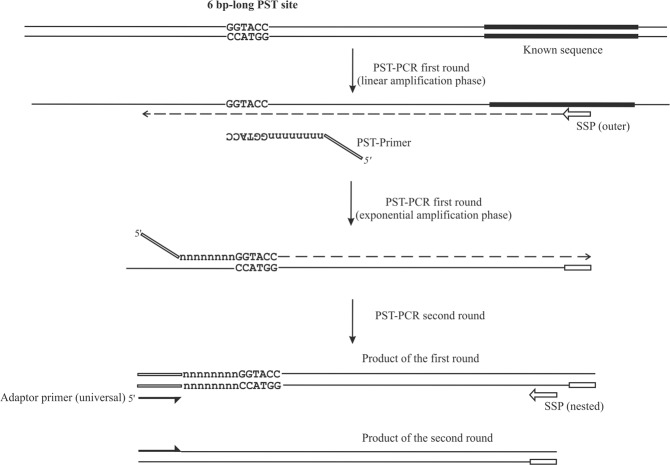
Schematic depiction of the PST-PCR method. The figure shows two consecutive PCR rounds. The first round is performed with one sequence-specific primer (SSP) and one PST primer. The SSP anneals to a target with a known sequence. The PST primer anchors to a palindromic sequence in a region with an unknown sequence. The second round is performed with one tail primer and one SSP. The regions with known sequences are depicted with thick lines. Other regions of the template with unknown sequences are shown as thin lines.

The PST-PCR process is schematically depicted in Fig. 1. The cornerstone of the PST-PCR technology is a specific design of primers, the PST primers, which are capable of annealing to uncharacterized sequences. A PST primer has a defined palindromic sequence at the 3′-end, a sufficiently long degenerate sequence to allow certain primer species to anneal by base-pairing to 15–18 nt of contiguous sequence, and an adaptor region at the 5′-end. We call the palindrome together with the degenerate sequence the “core” sequence. The core sequence is used to calculate the primer T_m_. The adaptor sequence is 19 nt and invariable (Table 1).

**Table 1:** Primers for PST-PCR. Abbreviations: T_m_, melting temperature; CG% (percentage of C and G) calculated for the core sequence (3′-end palindrome and 10 degenerate positions) at primer concentration of 0.5 µM in the presence of 2 mM Mg^2+^; for the tail primer, calculations were made for 0.25 µM primer in absence of Mg^2+^; LC (%) - Linguistic Complexity calculated for 6-nt restriction sites.

ID	Sequence (5′-3′)	Restriction site	Tm (°C)	CG (%)	LC (%)
**Tail primer (for second round PCR)**					
5600	GTTGCGGCAGGTCCTCACC	—	69.1	68.1	89
**PST primers (for first round PCR)**					
5601	GTTGCGGCAGGTCCTCACCnnnnnnnnnnGACGTC	AatII	49.3	56.3	100
5602	GTTGCGGCAGGTCCTCACCnnnnnnnnnnAACGTT	AclI	46.1	43.8	100
5603	GTTGCGGCAGGTCCTCACCnnnnnnnnnnTTCGAA	AsuII	45.6	43.8	100
5604	GTTGCGGCAGGTCCTCACCnnnnnnnnnnTGGCCA	BalI	51.0	56.3	100
5605	GTTGCGGCAGGTCCTCACCnnnnnnnnnnGGATCC	BamHI	48.3	56.3	100
5606	GTTGCGGCAGGTCCTCACCnnnnnnnnnnTGATCA	BclI	44.7	43.8	100
5326	GTTGCGGCAGGTCCTCACCnnnnnnnnnnAGATCT	BglII	43.7	43.8	100
5607	GTTGCGGCAGGTCCTCACCnnnnnnnnnnATCGAT	ClaI	44.8	43.8	89
5608	GTTGCGGCAGGTCCTCACCnnnnnnnnnnGAATTC	EcoRI	43.7	43.8	100
5609	GTTGCGGCAGGTCCTCACCnnnnnnnnnnGATATC	EcoRV	42.3	43.8	89
5610	GTTGCGGCAGGTCCTCACCnnnnnnnnnnAAGCTT	HindIII	45.5	43.8	100
5611	GTTGCGGCAGGTCCTCACCnnnnnnnnnnGTTAAC	HpaI	43.6	43.8	100
5612	GTTGCGGCAGGTCCTCACCnnnnnnnnnnGGTACC	KpnI	48.2	56.3	100
5613	GTTGCGGCAGGTCCTCACCnnnnnnnnnnCCATGG	NcoI	49.1	56.3	100
5614	GTTGCGGCAGGTCCTCACCnnnnnnnnnnGCTAGC	NheI	49.3	56.3	89
5615	GTTGCGGCAGGTCCTCACCnnnnnnnnnnCACGTG	PmaCI	50.2	56.3	100
5616	GTTGCGGCAGGTCCTCACCnnnnnnnnnnCTGCAG	PstI	49.7	56.3	100
5617	GTTGCGGCAGGTCCTCACCnnnnnnnnnnCAGCTG	PvuII	49.7	56.3	100
5618	GTTGCGGCAGGTCCTCACCnnnnnnnnnnGAGCTC	SacI	48.8	56.3	100
5619	GTTGCGGCAGGTCCTCACCnnnnnnnnnnGTCGAC	SalI	49.3	56.3	100
5620	GTTGCGGCAGGTCCTCACCnnnnnnnnnnAGTACT	ScaI	43.7	43.8	100
5621	GTTGCGGCAGGTCCTCACCnnnnnnnnnnGCATGC	SphI	50.9	56.3	89
5622	GTTGCGGCAGGTCCTCACCnnnnnnnnnnAGGCCT	StuI	50.1	56.3	100
5327	GTTGCGGCAGGTCCTCACCnnnnnnnnnnTCTAGA	XbaI	43.1	43.8	100
5623	GTTGCGGCAGGTCCTCACCnnnnnnnnnnCTCGAG	XhoI	48.6	56.3	100

**Table 2:** Cycling conditions for PST-PCR.

Reaction type	Number of cycles	Thermal conditions
**First round**		
		95 °C (2 min)
Linear	7–18	95 °C (15 sec), 65–72 °C (20 sec), 72 °C (1 min)
Exponential	12–18	95 °C (15 sec), 50–60 °C (15 sec), 72 °C (1 min)
Dilution 5–10 times		
**Second round**		
		95 °C (1 min)
Exponential	28–32	95 °C (15 sec), 68–72 °C (70 sec)
		72 °C (2 min)

Much like any other approach that utilizes PCR methodology, the sensitivity and specificity of PST-PCR are controlled by the thermal cycling profile; annealing temperatures play paramount roles. In this study we present the thermal profile (Table 2) that an interested researcher may consider as starting conditions for development of customized protocols. The thermal profile of the first round includes two phases: a linear amplification (driven by an SSP only) and an exponential amplification (involving both SSP and PST primers). The idea is that the PST primer begins participating in the PCR only after significant amounts of SSP-primed linear templates have accumulated, such that annealing to a PST site is thermodynamically favored.

In PST-PCR, the thermal cycling profile allows fine tuning of the balance between sensitivity and specificity by switching from the specificity-favoring annealing temperature (68–72 °C) to the sensitivity-favoring annealing temperature (55–65 °C). To control the balance, a cycle number is determined after which the annealing temperature is reduced to switch the PCR from linear to exponential amplification. The authors recommend performing 7–18 cycles at a high annealing temperature and 12–18 cycles at a reduced annealing temperature. The threshold-cycle-number control allows easy optimization of the protocol for a particular target. To expedite the process, the recommended thermal cycling profile for the second-round PCR consists of only two steps (denaturing and combined annealing/extension). The entire PST-PCR protocol is very rapid and requires two to three hours. Moreover, with some targets it appeared that the first round alone already generated sufficient GW product and the second round was unnecessary (data not shown).

The ability of the PST-PCR method to provide fragments suitable for sequencing and short enough to be amplifiable depends on occurrence of particular PST sites in a template. As the SSPs are often targeted to the ends of known sequences, in the best case almost the whole GW product will represent the unknown DNA. Considering the practical limit for the majority of thermophilic polymerases to be 3000 bp, for PST-PCR to be successful, one would require the presence of a PST site within this distance from the SSP site. Given that many genomes or long genomic regions (such as chromosomes) have already been sequenced and assembled, in many cases it is possible to compute or well estimate mean distances between PST sites in whole genomes or regions thereof. A quick approximation of an arithmetic mean of expected lengths of PST-PCR products is a half of the mean distance between restriction sites for a relevant restriction enzyme in a given genome. For the convenience of researchers interested in applying PST-PCR, a table is provided that lists the mean distances between palindromes of 6 nt or 8 nt in a variety of genomes (Table S1). The table is helpful for selecting the PST sites that have the maximum probability of amplifying PST-PCR products matching the desired length.

The PST-PCR thermal cycling profile defines performance of the method as it controls the balance between specificity and sensitivity. By the presence of a linear amplification phase, our method resembles POP, SWPOP and the protocol of the APAgene GW kit. As described above, the linear amplification serves to increase the amount of templates to which a PST primer can anneal and that performance of the method can be controlled by changing the amount of cycles in the linear amplification. The other feature that may contribute to specificity is the possible formation of a hairpin loop at the 3′-end of the PST primer. The hairpin would prevent annealing of the 3′-end to a template outside of a PST site. However, the formation of hairpins may be detrimental to sensitivity and general PCR efficiency, as extension of the hairpins will result in primer self-dimers that will consume the PST primers and inhibit amplification of a target template. Our thermodynamic calculations (Table S2) show that with a primer concentration 0.5 µM, 2 mM Mg^2+^, and an annealing temperature 55–65 °C, 3′-terminal hairpins in the PST primers are unfavorable. Moreover, we believe that the presence of defined palindromes at the 3′-ends of the PST primers is beneficial, as the recommended annealing temperatures favor specific annealing of the palindromes to the PST sites. Base-pairing between the palindromic sequences of two PST primers is possible for the 3′-terminal palindromes at low temperatures. Pairing of such primers during PCR would result in formation of primer dimers that would inhibit the PCR. The authors recommend using a “hot start” to disrupt the dimers before amplification. The PST primers have a significant number of degenerate positions (10 positions in this study). In the authors’ experience, the PST primers work at the recommended high annealing temperatures. Furthermore, an optimal working concentration of the PST primers is 0.5 µM, as further increasing the working concentration did not result in higher yields of GW products. Rather, RAPD-like amplicons appeared.

In theory, PST-PCR can generate non-specific amplification product, consisting of amplified domains that are surrounded by PST sites in a particular template. However, in practice the authors have never encountered this kind of product (flanked by PST primers at both ends). This is probably because, during PST-PCR, a specific GW product outcompetes side products, possibly due to annealing of an SSP being faster than annealing of a PST primer (due to the degeneracy of the latter). Therefore, the PCR products carrying the SSP primer at one end and the PST primer at the other end amplify more efficiently than products that have the PST primer at both ends. For any particular task, we recommend screening the PST primers to increase the probability of success. For example, for one template and an SSP, it is advisable to test all combinations of the SSP + PST primers listed in Table 1. Upon completion of a series of PST-PCR amplifications (one or two rounds), the researcher must analyze the PST-PCR products and select the reactions that contain one major product or a low number of products with a major species. This is our general approach for performing PST-PCR.

In the authors’ experience, different PST primers showed varying performance, and the best PST primers were those that had 6-bp palindromic sequences with an average CG content of 33–67%. For any GW task, we advise performing an initial screening to test as many PST primers (Table 1) as possible (no less than four) to maximize the probability of finding a working PST site. Not surprisingly, PST-PCR efficiency will depend on correctly designed SSPs. For multi-copy targets, such as ribosomal genes, successful PST-PCR capture was achieved using just one SSP primer that worked in both PCR rounds (data not shown). However, for rare variants and single-copy genes, it is preferable to develop nested SSPs. Additionally, the particular DNA polymerase employed affects the effectiveness of this approach at all stages. We recommend using DNA polymerases without proofreading ability (3′ → 5′ exonuclease activity).

### Quick troubleshooting

For the convenience of potential users of PST-PCR, in this section we discuss a number of problems that have been encountered with PST-PCR and their solutions. For some practical tasks, several changes to the overall procedure were made to increase quality of template or to improve PCR efficiency.

*1*. *No PCR products appear after second-round PCR with any PST primers* (Table 1), *even after increasing the number of cycles in the second round from 32 to 35*.

- In most cases, this is due to a problem with SSP design, leading to poor SSP annealing. Other causes may be insufficient template quality (genomic DNA) or problems related to general PCR setup, such as reaction mixture composition. Try a different SSP and verify the quality of the template and PCR reagents by performing a control PCR with a qualified template and reagents.
- Reduce the annealing temperatures of the first-round PCR by few degrees. For example, reduce the temperature from 68 °C to 65 °C during the linear amplification phase, and from 55 °C to 50 °C during the exponential amplification phase.
- Use more template and more units of Taq-polymerase and increase primer concentrations in the reaction mixture.

2. *Multiple products are observed after second-round PCR with any PST primer*.

- For a single-copy target, the appearance of multiple products is often due to SSP design. If combinations with many PST primers were attempted and multiple products appear in every reaction, this is because the SSP does not produce PCR products that amplify with sufficient efficiency to outcompete the side products. The solution here is to redesign the SSP, in particular to target the SSP to a different location in the known sequence. However, for multi-copy targets such as certain genes, repetitious sequences, or transposable elements, the detection of multiple bands is the correct and expected result, which represents GW products from targets in different genomic locations.
- In more than half the cases, single major bands will be observed with each PST primer. In some cases, more than one band may be observed for multi-copy sequences. The exact size of the bands will depend on the positions of restriction sites from the site-specific primer. Typically, secondary PCR products will range from 100 bp to 3 kb. In our experience, the product is not observed for some PST primers. This usually happens when the PST primer does not anneal efficiently near the target site.

### Frequencies of palindromes in eukaryotic genomes

The frequencies of 6 bp and 8 bp PST sites in selected eukaryotic genomes were calculated and appeared to differ (Table S1). The mean distance between the 6 bp PST sites ranges from 1–30 kb and depends mainly on GC content, whereas between the 8 bp PST sites the distances range from 4–15 kb. In general, the GC-rich sites are rare in eukaryotic genomes and the AT-rich sites occur more frequently. The GC-rich 8-bp sites are extremely rare (Table S1). In case the researcher needs to design new PST primers that are not listed in Table 1, the authors suggest avoiding very rare or very frequent PST sites. Useful calculated distances between the PST sites are given in Table S1.

### PST-PCR capture of uncharacterized sequences from the *VRN1* gene

The *VERNALIZATION 1* (*VRN1*) gene was selected as a model gene to test the efficiency of the PST-PCR approach because this gene is an important genetic marker in cereals. Sequences of the *P*. *pratense VRN1* gene promoter and introns have not been published before. The *VRN1* gene is present as a single copy per haploid genome; thus, the gene is a good example of a low-copy-number target. Although GW techniques are believed to be better suited to multi-copy targets^23^, here PST-PCR successfully captured a single-copy target.

**Figure 2:**
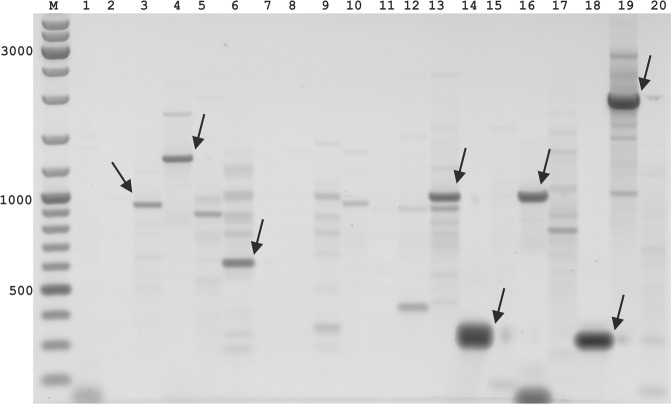
Results of PST-PCR gene walking in the *VRN1* gene. Products of the second-round PCR were analyzed by gel electrophoresis. Gels were stained with EtBr and scanned with an FLA-5100 imaging system (Fuji Photo Film GmbH) at a resolution of 50 µm. Arrows indicate the bands extracted for cloning and sequencing. Primers used in the first round were 5445 (Table 2) paired with one of the PST primers (1–17: 5601–5617; 18–20: 5621–5623, Table 2). Primers used in the second round were 5446 and 5600 (Table 2).

Initially, an analysis of sequence conservation was performed for exons of *VRN1* genes from representatives of the *Poaceae* family. Evolutionarily conserved regions were found in accordance to previously published works^24–26^. A set of outer and nested SSPs was designed (Tables 3, S4) to target exons 1, 2, and 8 and the promoter region. The SSPs were paired with PST primers (Table 1) to test all possible combinations of the outer SSP + PST primer. PCR products of various lengths were generated in the PST-PCR (see example gel in Fig. 2). All major bands were excised from the gel for cloning and sequencing. Sequencing was performed using the Sanger method. Indeed, all major bands appeared to be desired products of GW in the *VRN1* gene. The sequencing resulted in identification of the *VRN1* gene’s full-length promoter and intron 1. The sequences were deposited in Genbank (MK007525, MK240190-MK240221).

**Table 3:** Sequence-specific primers (SSPs) for the *VRN1* gene from *Poaceae*.

ID	Sequence (5′-3′)	Information	T_m_ (°C)	CG (%)	LC (%)	*Lolium perenne*, DNA (JN969603)	*Lolium perenne*, mRNA (AY198326)
5315	CTSAAGCGGATCGAGAACAAGATCAACC	F, exon1	61.5	50.0	78	185→212	
5410	CTCATCATCTTCTCCACCAAGGGAAAGCTCTACGAGTTC	F, exon1	66.2	48.7	81	296→334	
5299	GTTCTCGATCCGCTTSAGCTGCACCTT	R, exon1	64.5	55.6	89	176←202	
5411	GCACGGAGATCTCGTGCGCCTTCTTGAG	R, exon1	66.8	60.7	89	248←275	
5412	CTCGTAGAGCTTTCCCTTGGTGGAGAAGATGATGAG	R, exon1	65.3	50.0	80	296←331	
5317	ARCGGTAYGAGCGYTACTCYTATGCAGA	F, exon2	62.3	50.0	87	14265→14292	366→393
5413	GARCGGTATGAGCGCTAYTCYTATGCAGA	F, exon2	62.4	50.0	83	14264→14292	365→393
5300	TARGAGTARCGCTCRTACCGYTCAAGAA	R, exon2	60.5	46.4	83	14259←14286	360←387
5301	GTARCGCTCRTACCGYTCAAGAATTTTGTCCATA	R, exon2	62.8	42.6	88	14248←14281	349←382
5416	CAGCCGTTGATGTGGCTCACCATCCA	R, exon8	64.7	57.7	93	16421←16441	872←897
5445	CTTGTTTTGGGCCGTCTCGCTTC	R, promoter	61.2	56.5	73	promoter primer	
5446	CGTCTCGCTTCTCCCGTTTGGGCAT	R, promoter	64.9	60.0	81		

Abbreviations: T_m_ melting temperature, calculated for a primer concentration of 0.25 µM in the absence of Mg^2+^; CG% percentage of C and G; LC (%) - Linguistic Complexity calculated for 6-nt restriction sites.

### PST-PCR will have applications beyond genome walking

Genomic fingerprinting for various identification purposes has become a routine procedure to control production, transportation, and consumption of plants and plant-derived products. New molecular genetic methods for comparison of whole genomes or identification of selected genetic markers are continually being developed and compete with each other for their informative capacity, costs, and other properties^4,8,11,17,20^. For this purpose, the PST-PCR method is worth also considering.

**Figure 3:**
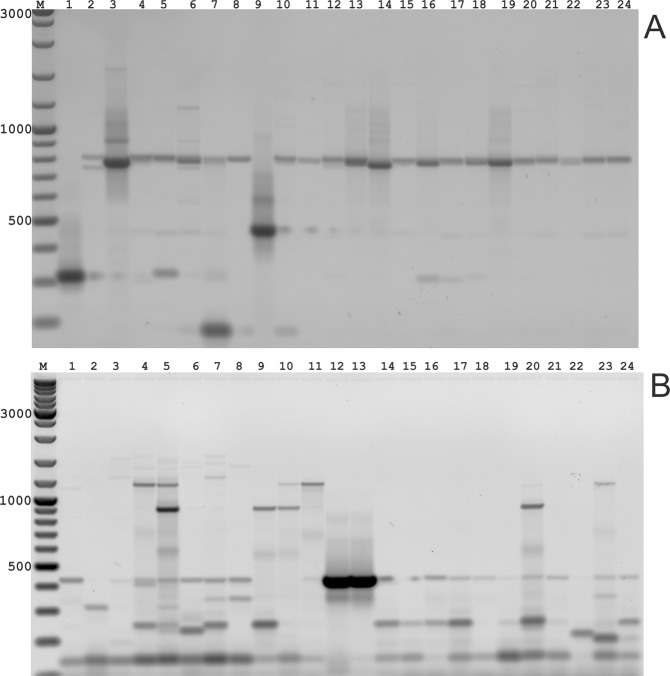
Gel electrophoresis of second-round PCR products to analyze sequence polymorphisms in *VRN1* intron 1 (**a**) and the *VRN1* promoter (**b**) between Timothy-grass plants. Lanes contain the following Timothy-grass samples: 1 - 251595 (former Yugoslavia); 2 - 319079 (Spain); 3 - Kew 6091 (Italy); 4 - 381926 (France); 5 - Kew 51998 (England); 6 - Kew 6116 (Italy); 7 - 3199 (Russia); 8 - 3264 (Ireland); 9 - 325461 (Russia); 10 - 210426 (Greece); 11 - Grindstad; 12 - RCAT41183 (Hungary); 13 - A7E0001 (Bulgaria); 14 - BORN21/1 (Finland); 15 - 3267 (Romania); 16 - 319080 (Spain); 17 - 539037 (Russia); 18 - BOR0307 (Finland); 19 - BOR112 (Finland); 20 - NGB7596 (Norway); 21 - NGB1332 (Sweden); 22 - NGB4140 (Iceland); 23 - 14G2400152 (Slovakia); 24 - 325461 (Russia).

The results presented in this study allow us to propose PST-PCR as a method for species identification, for example, in the *Poaceae* using the *VRN1* gene as a target. In this regard, a multiple alignment of the *VRN1* gene exon 1 shows regions of varying conservation suitable for discrimination of all *Poaceae* species (Table S4, Figure S2). The same primers used in the PST-PCR with the *P*. *pratense* genome were subsequently tested with the *Lolium perenne* and *Festulolium spp*. Genomes; these also generated GW fragments (publication in preparation). The *VRN1* gene has the potential to be a universal marker for fingerprinting of cereals and grasses. Alignment of DNA sequences of the *VRN1* exons from various members of *Poaceae* revealed both conserved and variable regions. Primers targeting exon 1 were tested on all representatives from *Phleum pratense* available to the authors and universally led to amplification of the desired PCR product. DNA sequencing revealed the existence of sequence polymorphisms in the promoter and intron 1 (Fig. 3). Analysis of the molecular organization of the gene, including the lengths of the promoter and intron 1 and copy-number variation, allowed description of polymorphisms suitable for fingerprinting of all *Poaceae* species. The protocols described herein may be used in the identification of lines and intraspecific and inter-species genetic variability in members of the *Poaceae*. Using the described PST-PCR method, the authors are currently developing a high-throughput fingerprinting platform for plant genomes.

Possible applications of PST-PCR include population studies such as biodiversity monitoring. For this purpose, it is possible to SSP-target transposable elements such as long interspersed nuclear elements (LINEs), short interspersed nuclear elements (SINEs), LTR-retrotransposons, and DNA transposons^27^. Transposable elements are multi-copy targets and at least parts of their sequences are relatively conserved, thus simplifying selection of the SSPs.

Furthermore, PST-PCR is applicable to a broader scope of applications, including whole genome amplification, Amplified Fragment Length Polymorphism (AFLP)/Sequence-Specific Amplified Polymorphism (SSAP) multiple banding pattern techniques^28^, and locating the position of a genetically modified organism (GMO) transgene insertion by capture of a region adjacent to the left border of the transfer DNA (T-DNA)^18^. Locating genomic positions of transgene inserts in presumably GMO genomes is of importance in research projects as well as for various regulatory authorities. This may be achieved by utilizing known conserved transgene sequences or T-DNA fragments that can be targeted by SSPs in a PST-PCR approach^18^.

Nevertheless, for fingerprinting and transgene detection, several technical considerations for PST-PCR should be mentioned. The first is careful selection of SSPs. The stochastic nature of individual PCR reactions and PCR bias stemming from primer-template mismatches dictate that the optimal number of PCR cycles must be determined experimentally. Multiple annealing temperatures also should be tested to avoid dropouts (missing amplicons despite the existing targets). The second issue is the generation of potential false positives (off-target amplicons). Synthesis of side products is observed with any GW method. No GW approach so far described can in all cases avoid generation of nonspecific PCR products, although the sources of nonspecific amplification can be discriminated against by using combinations of nested primers. The PST-PCR method is, however, notable in that the generation of nonspecific PCR products is low. The authors provide a list of 30 PST primers whose ability for highly specific GW has been experimentally confirmed.

## Conclusions

We have described a novel PCR-based technique for capturing unknown sequences from whole-genome templates. We call this method palindromic sequence-targeted PCR (PST-PCR), which uses a distinctive design of walking primers in combination with sequence-specific primers. The defining features of the method are the design of PST primers and the thermal profile. The PST primers have a palindromic sequence at the 3′ end, a fully degenerate sequence, and a defined 5′-terminal adapter. The thermal cycling profile has a liner amplification phase and an exponential amplification phase. The PST-PCR method is rapid and demonstrated high sensitivity and specificity in experiments to capture the promoter and intron 1 of the *VRN1* gene from a plant genome.

## Methods

### Plant material and DNA extraction

Timothy-grass plants were collected from various geographic locations^29^. Leaves for DNA isolation were collected from 28-day-old plants grown in a greenhouse. Genomic DNA was extracted using a CTAB-based protocol and treated with RNase A. A detailed protocol for DNA isolation is deposited at protocols.io (DOI: 10.17504/protocols.io.mghc3t6). DNA samples were diluted in 1X TE buffer (10 mM Trizma pH 7.5, 1 mM EDTA) and DNA quality was verified using a Nanodrop spectrophotometer (Thermo Fisher Scientific) and gel electrophoresis.

### *In silico* genome analysis of PST site frequency

To demonstrate the generality of the PST-PCR method, the frequencies of PST sites were calculated for various commonly studied plant and animal genomes. Complete genomes were extracted from the NCBI database (https://www.ncbi.nlm.nih.gov/genome/browse/). Frequencies of PST sites (6 nt or 8 nt) were computed using FastPCR software (http://primerdigital.com/fastpcr.html)^30,31^. The number of PST sites per haploid genome and an average distance between the neighboring PST sites were computed and are presented in the Supplementary Material (Supplementary Dataset).

### PST primer design

The PST primers were designed to meet the following conditions: a 6-nt palindromic sequence is present at the 3′-end; a 10-nt fully degenerate sequence (dN_10_) is present upstream (i.e. in the direction of the 5′-terminus) of the palindrome; an adaptor region with a distinctive sequence is present upstream of the degenerate sequence. The invariable adaptor in this study was 19 nt (Table 1). The calculated T_m_ and GC content for the PST primers are presented in Table 1. The T_m_ were computed for the 16-nt “core” sequences (in this study we define the palindrome together with the degenerate sequence as “core” sequence), as only the core sequences of the PST primers are expected to anneal to template DNA. T_m_ was calculated using a nearest-neighbor thermodynamic model with the following reaction conditions: 50 mM monovalent cation (K^+^/NH_4_^+^), 2 mM Mg^2+^, and 0.5 μM of PST primer. Thermodynamic calculations and modeling of primer secondary structures were performed using FastPCR software^30–32^. Linguistic complexity (LC), a formal measure of informational content in a nucleotide sequence, was computed for palindromic sequences. As an important practical note, for any particular task we recommend screening PST primers. For example, in an experiment it is advisable to set up multiple PST-PCR amplifications that have the same template and SSP, together with different PST primers from Table 1.

### Sequence-specific primer selection

Sequences of *VERNALIZATION 1* (*VRN1*) genes from representative species in the *Poaceae* were downloaded from GenBank (a species list and accession numbers are presented in the Supplementary Materials). Sequences of individual exons of the *VRN1* genes were extracted and used to build multiple alignments using MULTALIN (https://prabi.ibcp.fr/htm/site/web/home)^33^. Regions within the exons that are highly conserved in all available *Poaceae VRN1* genes were used to target the SSPs. We designed two site-specific primers, one for the primary PCR reaction (SSP1) and the other one nested, annealing to sequences beyond the 3′ end of the primary PCR primer, for the secondary PCR reaction (SSP2). Sets of SSPs were designed to target the termini of the selected exons and one conserved region in the promoter region of the *VRN1* gene, which before this study had been determined for some species in the *Poaceae* family but not for *P*. *pratense*. The computed SSPs collectively guaranteed bidirectional GW to capture introns and unknown parts of the promoter. The SSPs are listed in Table 3.

The following rules were used for selection of the SSPs: each SSP should be 25–35 nt and have a GC content of 40–60%, and calculated T_m_ ≥65 °C. A good SSP should not be capable of forming self-dimers. Outer and nested primers preferably should not overlap. Generally, both the SSPs should be as close to the end of the known sequence as possible. The SSPs were designed using FastPCR software with the following calculation parameters: 50 mM monovalent cation (K^+^/NH_4_^+^), no divalent cations, a working primer concentration of 25 μM, and the software’s XCR option turned on.

### PST-PCR set up and parameters

The PST-PCR was performed as a two-round PCR. In the first round, various combinations of PST primers with the outer SSPs were used. Reaction mixtures were prepared of which each particular reaction contained one SSP (for every outer SSP from Table 3) and one PST primer (for every PST primer listed in Table 1). All first-round amplifications were performed using the same reaction conditions. Upon completion of the first round, the first-round reaction mixtures were diluted 1/6 with 1X TE buffer and added as templates to the second-round reaction mixtures. Primers in the second-round reactions were the SSPs (same as in the first round, or nested) paired with the universal tail primer (5600 in Table 1). The thermal cycling parameters for the two rounds of the PST-PCR process are shown in Table 2 and Figure S1.

### Example protocol for first-round PCR

The first-round PCR was performed in a 30-µL reaction mixture consisting of 30 ng template DNA, 1X LongAmp *Taq* reaction buffer, and 1 U LongAmp Taq DNA Polymerase (NEB). The reaction contained 2 mM Mg^2+^, 200 μM each dNTP, 0.2 μM SSP, and 0.5 μM PST primer. The first round used the following thermal profile: initial denaturation (95 °C for 2 min), then 18 cycles of linear amplification (95 °C for 15 s, 68 °C for 10 s, 72 °C for 60 s), then 18 cycles of exponential amplification (95 °C for 15 s, 52 or 55 °C for 10 s, 72 °C for 60 s. The final extension step was at 72 °C for 2 min. The reaction mixture was diluted 1/6 in 1X TE buffer in each PCR tube and used as a template in the second-round amplification.

### Example protocol for second-round PCR

The reaction mixtures (25 μl) consisted of 1X LongAmp *Taq* reaction buffer, 1 U LongAmp Taq DNA Polymerase (NEB), 0.2 μM SSP, and 0.2 μM tail primer. A total of 2 μl of the diluted product from the first round was added to the reaction mixtures described above. The thermal cycling profile was 95 °C for 1 min (initial denaturation), then 28–30 two-step cycles (95 °C for 15 sec, 70 °C for 90 sec), and a final extension at 72 °C for 2 min. The PCR products were separated by electrophoresis at 70–90 V for 3 hours in a 1.2% agarose gel (Wide Range, SERVA Electrophoresis GmbH) in 0.5X TBE electrophoresis buffer. Gels were stained with EtBr and scanned using an FLA-5100 imaging system (Fuji Photo Film GmbH) at a resolution of 50 µm. Selected PCR products were cloned for sequencing.

## Supplementary information

Suplemental Material

Supplementary Dataset

## Data Availability

Accession numbers for the sequences resulting from this study: MK007525, MK240190-MK240221.
