# Supplementary material for: Palindromic sequence-targeted (PST) PCR: a rapid and efficient method for high-throughput gene characterization and genome walking
Source: Sci Rep. 2019 Nov 27;9:17707. doi: 10.1038/s41598-019-54168-0 (PMC6881309; doi:10.1038/s41598-019-54168-0)
Supplement: Supplementary file 1 — Suplemental Material [file 41598_2019_54168_MOESM1_ESM.pdf]

Supplementary Information

Palindromic sequence-targeted (PST) PCR: a rapid and efficient method for high-throughput gene characterization and genome walking

Ruslan Kalendar[1,2*], Alexandr V. Shustov[3], Mervi M. Seppänen[1], Alan H. Schulman[4,5], Frederick L. Stoddard[1]

[1] Department of Agricultural Sciences, Viikki Plant Science Centre and Helsinki Sustainability Centre, University of Helsinki, P.O. Box 27 (Latokartanonkaari 5), FI-00014 Helsinki, Finland

[2] PrimerDigital Ltd, FIN-00710, Helsinki, Finland

[3] National Center for Biotechnology, Korgalzhin hwy 13/5, 010000 Astana, Kazakhstan

[4] Institute of Biotechnology and Viikki Plant Science Centre, University of Helsinki, P.O. Box 65, FI-00014 Helsinki, Finland

[5] Natural Resources Institute Finland (Luke), Latokartanonkaari 9, FI-00790 Helsinki, Finland

Supplementary Table S1. Average distances between neighboring restriction sites (calculated from genome-wide analysis).

**Restriction enzyme**	**Sequence (5'-3')**	**CG (%)**	**LC (%)**	**Average distance (bp) between neighboring restriction sites in the listed genomes**						
				**Average**	***Homo sapiens***	***Bos taurus***	***Capra hircus***	***Arabidopsis thaliana***	***Medicago truncatula***	***Capsicum annuum***
-	AAATTT	0.0	56	**918**	1,283	1,174	1,108	787	589	746
**AclI**	**AACGTT**	**33.3**	**100**	**13,120**	**18,005**	**22,355**	**20,049**	**4,338**	**6,522**	**13,834**
**HindIII**	**AAGCTT**	**33.3**	**100**	**2,944**	**3,813**	**3,671**	**3,422**	**1,794**	**2,712**	**2,838**
**SspI**	AATATT	0.0	67	**1,092**	1,449	1,190	1,140	1,011	737	960
**BspLU11I**	ACATGT	33.3	100	**2,716**	3,104	3,166	3,084	2,557	2,147	2,674
**AgeI**	ACCGGT	66.7	100	**33,471**	58,537	70,217	63,959	8,056	12,163	28,565
**MluI**	ACGCGT	66.7	89	**76,212**	150,974	168,506	138,438	22,844	35,766	59,234
**SpeI**	ACTAGT	33.3	100	**5,066**	8,121	6,717	6,281	4,769	4,763	3,243
**BglII**	**AGATCT**	**33.3**	**100**	**3,551**	**4,104**	**3,439**	**3,177**	**2,733**	**4,664**	**3,273**
**Eco47III**	AGCGCT	66.7	89	**30,753**	20,585	31,032	25,966	25,251	33,245	44,232
**StuI**	**AGGCCT**	**66.7**	**100**	**7,314**	**3,344**	**3,962**	**3,605**	**12,079**	**12,274**	**7,524**
**ScaI**	**AGTACT**	**33.3**	**100**	**4,382**	**5,765**	**3,433**	**3,432**	**5,100**	**5,392**	**3,645**
**ClaI**	**ATCGAT**	**33.3**	**89**	**16,568**	**37,812**	**41,648**	**37,919**	**3,839**	**6,320**	**5,430**
**AvaIII**	ATGCAT	33.3	89	**2,396**	3,655	2,954	2,943	2,384	1,919	1,811
**VspI**	ATTAAT	0.0	67	**1,597**	2,284	2,148	2,051	1,285	932	1,313
**MfeI**	CAATTG	33.3	100	**3,955**	5,880	5,869	5,567	3,245	2,348	3,095
**PmaCI**	**CACGTG**	**66.7**	**100**	**11,547**	**10,869**	**12,275**	**11,827**	**8,355**	**9,437**	**16,558**
**PvuII**	**CAGCTG**	**66.7**	**100**	**7,185**	**2,758**	**3,399**	**3,088**	**6,132**	**9,505**	**11,790**
**NdeI**	CATATG	33.3	89	**2,976**	3,691	3,750	3,598	3,017	2,978	2,428
**NcoI**	**CCATGG**	**66.7**	**100**	**4,566**	**4,112**	**1,659**	**1,677**	**5,710**	**7,321**	**5,264**
**SmaI**	CCCGGG	100.0	56	**34,215**	7,721	18,551	16,323	42,378	55,967	46,414
**SacII**	CCGCGG	100.0	67	**71,156**	43,124	74,494	62,264	28,544	71,714	131,969
**AvrII**	CCTAGG	66.7	100	**8,712**	5,051	7,686	7,113	17,348	15,713	6,362
**PvuI**	CGATCG	66.7	89	**104,836**	234,202	314,063	261,034	12,470	35,721	24,191
**XmaIII**	CGGCCG	100.0	67	**74,839**	32,004	77,947	63,959	36,095	63,080	159,155
**SplI**	CGTACG	66.7	89	**119,215**	292,890	311,638	240,311	18,363	41,002	56,838
-	CTATAG	33.3	89	**5,150**	6,138	5,721	5,335	5,637	6,272	3,589
**XhoI**	**CTCGAG**	**66.7**	**100**	**16,419**	**24,755**	**32,080**	**28,834**	**6,942**	**17,529**	**10,616**
**PstI**	**CTGCAG**	**66.7**	**100**	**6,263**	**2,322**	**1,909**	**1,803**	**5,444**	**7,868**	**12,794**
**AflII**	CTTAAG	33.3	100	**4,125**	4,938	5,043	4,790	4,101	4,286	2,905
**EcoRI**	GAATTC	33.3	100	**3,246**	3,708	3,046	2,801	3,307	3,542	2,955
**AatII**	GACGTC	66.7	100	**28,202**	45,747	48,947	43,918	13,070	24,860	29,053
**SacI**	**GAGCTC**	**66.7**	**100**	**7,635**	**4,920**	**6,630**	**6,212**	**6,048**	**12,266**	**8,874**
**EcoRV**	**GATATC**	**33.3**	**89**	**5,095**	**7,657**	**7,087**	**6,582**	**3,772**	**4,756**	**3,324**

**SphI**	**GCATGC**	**66.7**	**89**	**6,545**	**5,527**	**5,798**	**5,330**	**9,365**	**7,387**	**6,937**
**KroI**	GCCGGC	100.0	67	**47,340**	23,269	39,527	35,221	27,071	42,416	107,621
**BsePI**	GCGCGC	100.0	44	**66,484**	39,473	75,504	59,510	121,711	61,170	98,324
**NheI**	GCTAGC	66.7	89	**11,715**	10,946	15,054	13,474	11,785	12,485	11,663
**BamHI**	**GGATCC**	**66.7**	**100**	**8,035**	**8,168**	**9,318**	**8,787**	**7,309**	**10,175**	**7,935**
**NarI**	GGCGCC	100.0	67	**34,015**	12,648	42,956	37,150	36,137	44,136	40,970
**ApaI**	GGGCCC	100.0	56	**20,402**	6,125	10,010	9,053	46,384	41,820	17,944
**KpnI**	**GGTACC**	**66.7**	**100**	**11,170**	**10,895**	**13,429**	**12,934**	**14,469**	**12,208**	**7,263**
**SnaI**	GTATAC	33.3	89	**6,477**	7,848	6,559	6,181	5,883	7,237	5,685
**SalI**	**GTCGAC**	**66.7**	**100**	**39,097**	**104,035**	**77,238**	**69,586**	**12,839**	**24,067**	**21,554**
**ApaLI**	GTGCAC	66.7	100	**8,905**	6,459	8,556	8,344	14,061	10,626	9,463
**HpaI**	**GTTAAC**	**33.3**	**100**	**6,812**	**8,363**	**8,114**	**7,583**	**4,761**	**3,786**	**6,520**
**-**	TAATTA	0.0	67	**1,586**	2,406	2,278	2,184	1,197	970	1,297
**SnaBI**	TACGTA	33.3	89	**15,782**	26,063	31,504	30,282	6,323	9,922	9,102
**-**	TAGCTA	33.3	89	**4,792**	5,279	6,668	6,160	4,240	5,455	3,691
**BspHI**	TCATGA	33.3	100	**2,456**	3,320	2,943	2,676	2,664	2,479	1,627
**BspMII**	TCCGGA	66.7	100	**25,551**	31,954	43,890	38,485	9,584	15,757	34,809
**NruI**	TCGCGA	66.7	89	**73,616**	218,191	156,727	131,170	21,383	24,150	37,195
**XbaI**	**TCTAGA**	**33.3**	**100**	**3,510**	**3,948**	**3,966**	**3,677**	**3,739**	**4,355**	**2,913**
**BclI**	**TGATCA**	**33.3**	**100**	**3,008**	**4,329**	**3,876**	**3,636**	**2,284**	**2,647**	**2,476**
**MstI**	TGCGCA	66.7	89	**31,020**	34,966	55,723	40,216	21,859	23,430	34,871
**BalI**	**TGGCCA**	**66.7**	**100**	**5,371**	**2,291**	**2,918**	**2,737**	**7,376**	**8,686**	**6,091**
**Bsp1407I**	TGTACA	33.3	100	**3,874**	3,960	3,344	3,139	4,037	3,734	4,129
**PsiI**	TTATAA	0.0	67	**1,624**	2,053	2,014	1,904	1,260	1,001	1,373
**AsuII**	**TTCGAA**	**33.3**	**100**	**13,117**	**27,922**	**30,112**	**27,958**	**3,671**	**4,779**	**6,031**
**-**	TTGCAA	33.3	100	**2,593**	3,329	2,874	2,668	2,346	1,993	2,665
**AhaIII**	TTTAAA	0.0	56	**972**	979	915	866	891	677	912
				**20,022**						

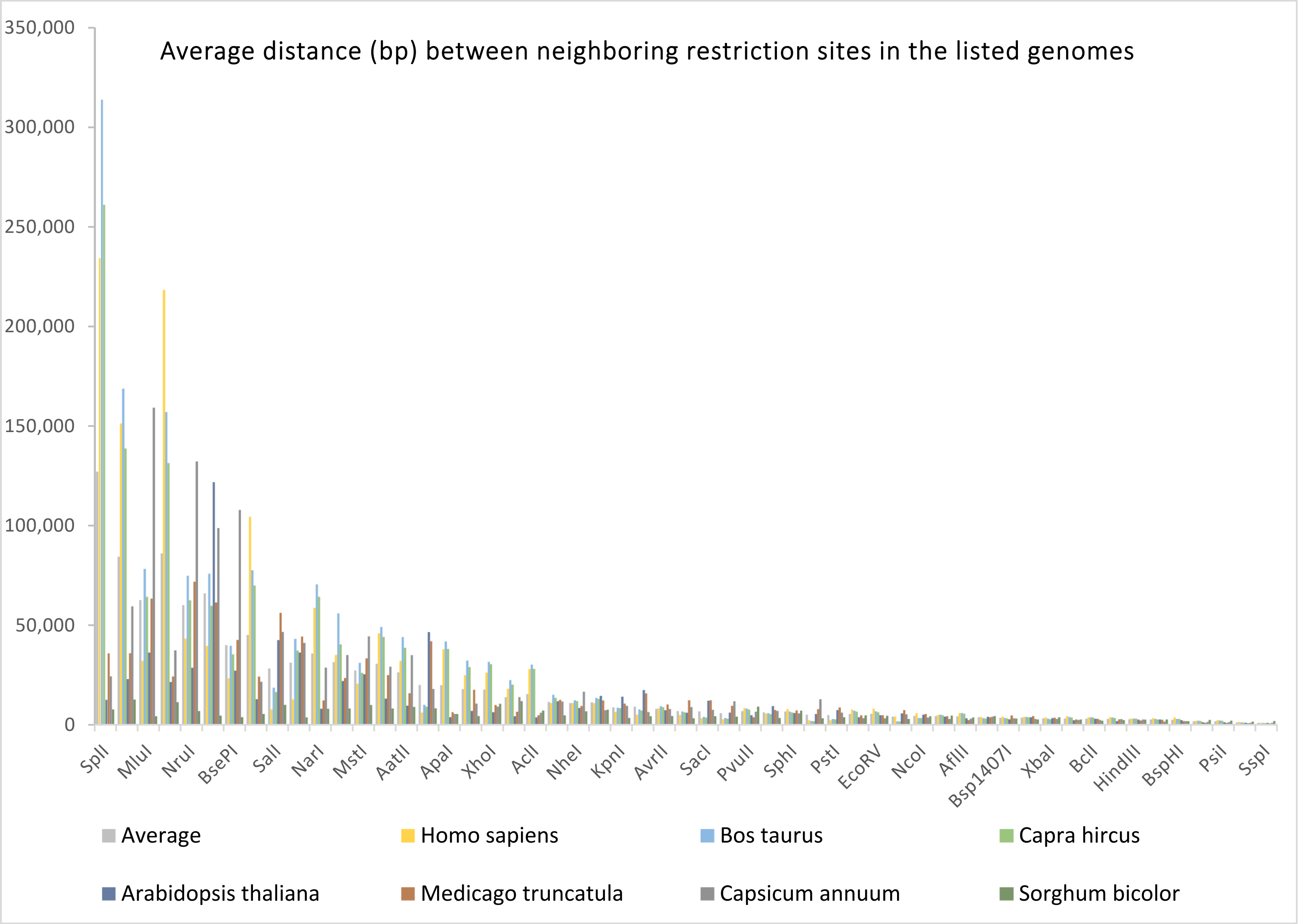
Average distance (bp) between neighboring restriction sites in the listed genomes
350,000
300,000
250,000
200,000
150,000
100,000
50,000
0
SplI
MluI
NruI
BsePI
SalI
NarI
MstI
AatII
ApaI
XhoI
AclI
NheI
KpnI
AvrII
SacI
PvuII
SphI
PstI
EcoRV
NcoI
AflII
Bsp1407I
XbaI
BclI
HindIII
BspHI
PsiI
SspI
Average
Homo sapiens
Bos taurus
Capra hircus
Arabidopsis thaliana
Medicago truncatula
Capsicum annuum
Sorghum bicolor

Supplementary Table S2. PST primers and T_m for self-dimers. Abbreviations: T_m melting temperature, calculated for 0.5 μM primer and 2 mM Mg^{2+}.

ID	Sequence (5'-3')	Restriction sites	T_m for self-dimer, °C
5601	GTTGCGGCAGGTCCTCACCnnnnnnnnnnGACGTC	AatII	10.5
5602	GTTGCGGCAGGTCCTCACCnnnnnnnnnnAACGTT	AclI	5.9
5603	GTTGCGGCAGGTCCTCACCnnnnnnnnnnTTCGAA	AsuII	-
5604	GTTGCGGCAGGTCCTCACCnnnnnnnnnnTGGCCA	BalI	16.8
5605	GTTGCGGCAGGTCCTCACCnnnnnnnnnnGGATCC	BamHI	4.5
5606	GTTGCGGCAGGTCCTCACCnnnnnnnnnnTGATCA	BclI	-
5326	GTTGCGGCAGGTCCTCACCnnnnnnnnnnAGATCT	BglII	-
5607	GTTGCGGCAGGTCCTCACCnnnnnnnnnnATCGAT	ClaI	-
5608	GTTGCGGCAGGTCCTCACCnnnnnnnnnnGAATTC	EcoRI	-
5609	GTTGCGGCAGGTCCTCACCnnnnnnnnnnGATATC	EcoRV	-
5610	GTTGCGGCAGGTCCTCACCnnnnnnnnnnAAGCTT	HindIII	-
5611	GTTGCGGCAGGTCCTCACCnnnnnnnnnnGTTAAC	HpaI	-
5612	GTTGCGGCAGGTCCTCACCnnnnnnnnnnGGTACC	KpnI	4.7
5613	GTTGCGGCAGGTCCTCACCnnnnnnnnnnCCATGG	NcoI	6.7
5614	GTTGCGGCAGGTCCTCACCnnnnnnnnnnGCTAGC	NheI	9.3
5615	GTTGCGGCAGGTCCTCACCnnnnnnnnnnCACGTG	PmaCI	12.5
5616	GTTGCGGCAGGTCCTCACCnnnnnnnnnnCTGCAG	PstI	9.8
5617	GTTGCGGCAGGTCCTCACCnnnnnnnnnnCAGCTG	PvuII	9.8
5618	GTTGCGGCAGGTCCTCACCnnnnnnnnnnGAGCTC	SacI	7.7
5619	GTTGCGGCAGGTCCTCACCnnnnnnnnnnGTCGAC	SalI	10.5
5620	GTTGCGGCAGGTCCTCACCnnnnnnnnnnAGTACT	ScaI	-
5621	GTTGCGGCAGGTCCTCACCnnnnnnnnnnGCATGC	SphI	13.8
5622	GTTGCGGCAGGTCCTCACCnnnnnnnnnnAGGCCT	StuI	13.9
5327	GTTGCGGCAGGTCCTCACCnnnnnnnnnnTCTAGA	XbaI	-
5623	GTTGCGGCAGGTCCTCACCnnnnnnnnnnCTCGAG	XhoI	7.8

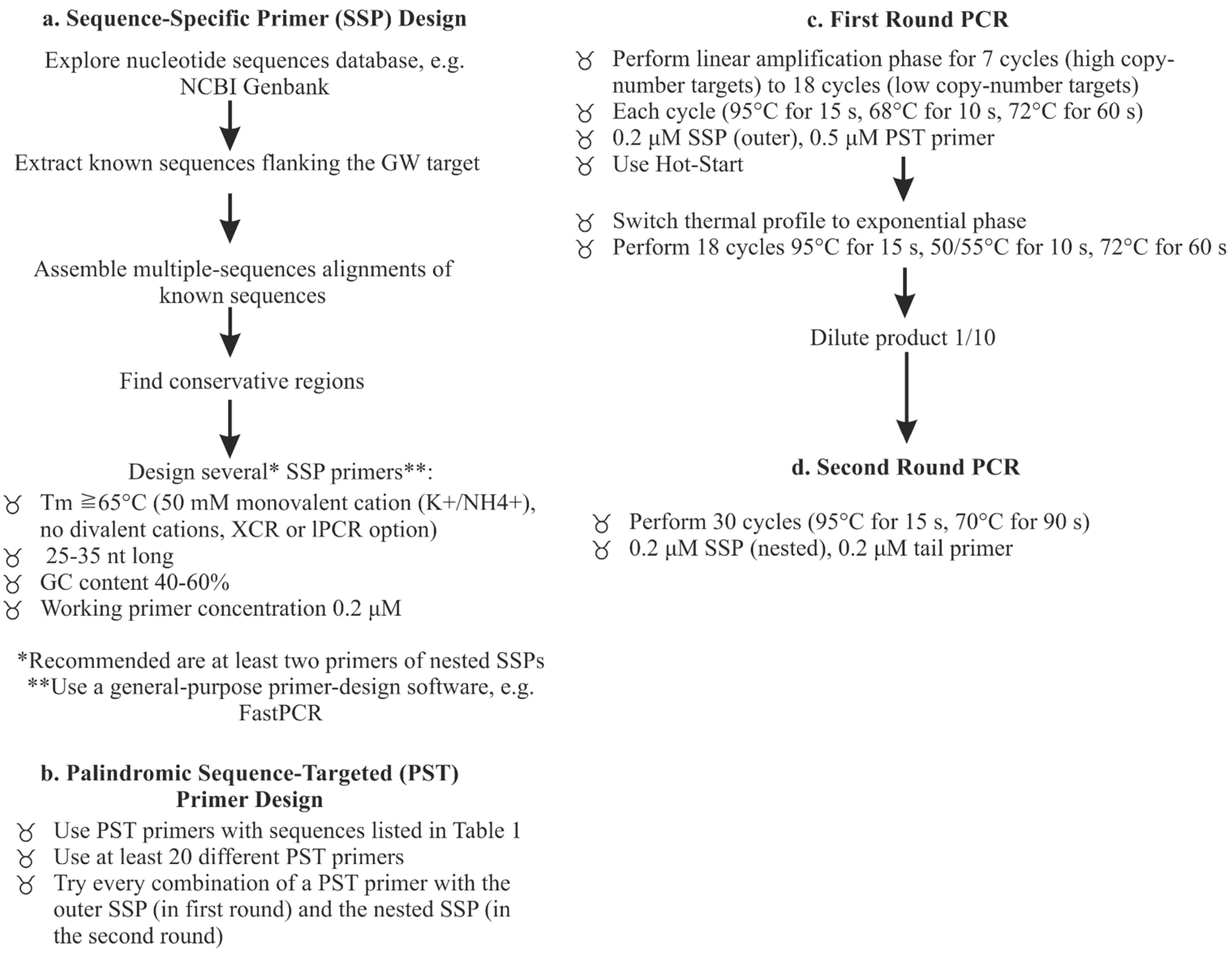

Supplementary Figure S1. Flowchart for primer design PST-PCR methodology.

Supplementary Table S3. Thermal conditions used in TAIL-PCR (LETAIL-PCR) method (Jia, X., Lin, X. & Chen, J. Linear and exponential TAIL-PCR: a method for efficient and quick amplification of flanking sequences adjacent to Tn5 transposon insertion sites. AMB Express 7, 195, doi:10.1186/s13568-017-0495-x (2017).)

Steps	Amplification style	Thermal condition	Dilution (fold)
1	Linear	98 °C 10 s, 62 °C 5 s, 72 °C 30 s; 20 cycles	100
	Exponential	98 °C 10 s, 25 °C 5 s, 72 °C 30 s; 1 cycle<br>98 °C 10 s, 58 °C 5 s, 72 °C 30 s; 18 cycles	100
2	Linear	98 °C 10 s, 62 °C 5 s, 72 °C 30 s; 20 cycles	10
	Exponential	98 °C 10 s, 68 °C 5 s, 72 °C 30 s, 98 °C 10 s, 63 °C 5 s, 72 °C 30 s, 98 °C 10 s, 50 °C 5 s, 72 °C 30 s; 7 cycles	100
3	Exponential	98 °C 10 s, 68 °C 5 s, 72 °C 30 s, 98 °C 10 s, 63 °C 5 s, 72 °C 30 s, 98 °C 10 s, 50 °C 5 s, 72 °C 30 s; 13 cycles	

Cycling conditions for PST-PCR

Reaction type	Number of cycles	Thermal conditions
First round		
		95°C (2 min)
Linear	7-18	95°C (15 sec), 65-72°C (20 sec), 72°C (1 min)
Exponential	12-18	95°C (15 sec), 50-60°C (10 sec), 72°C (1 min)
Dilution 5-10 times		
Second round		
		95°C (1 min)
Exponential	28-32	95°C (15 sec), 68-72°C (70 sec)
		72°C (2 min)

The Temperature Profile for the PST-PCR

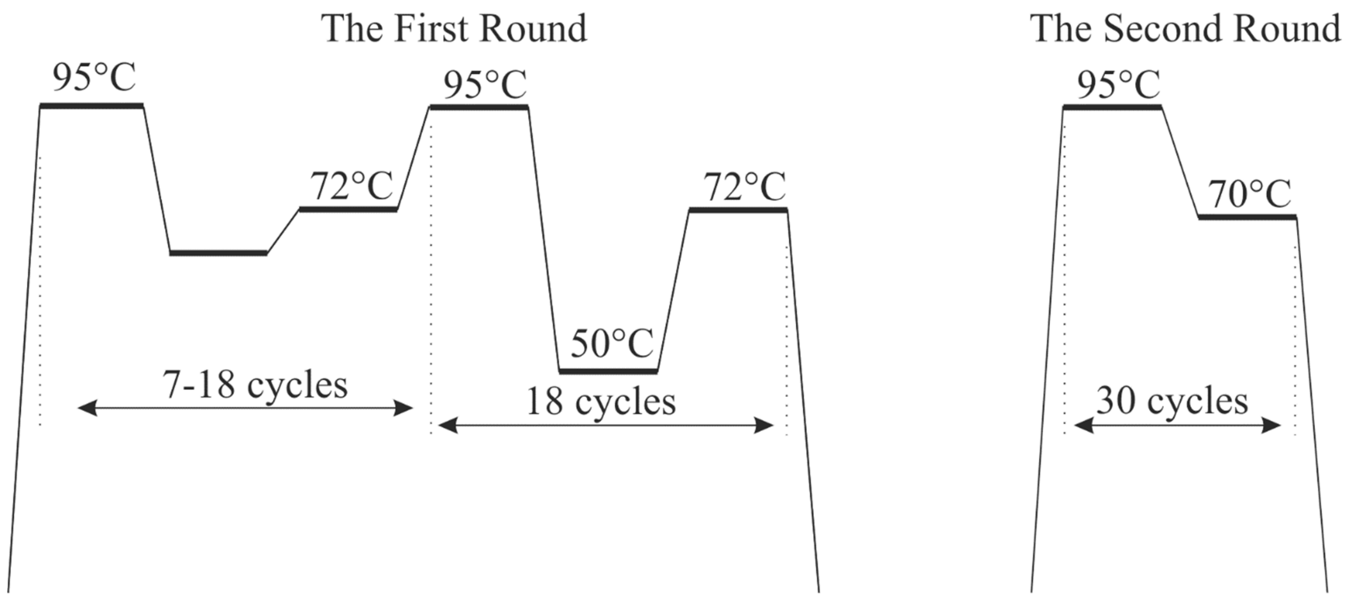

The Temperature Profile for the TAIL-PCR

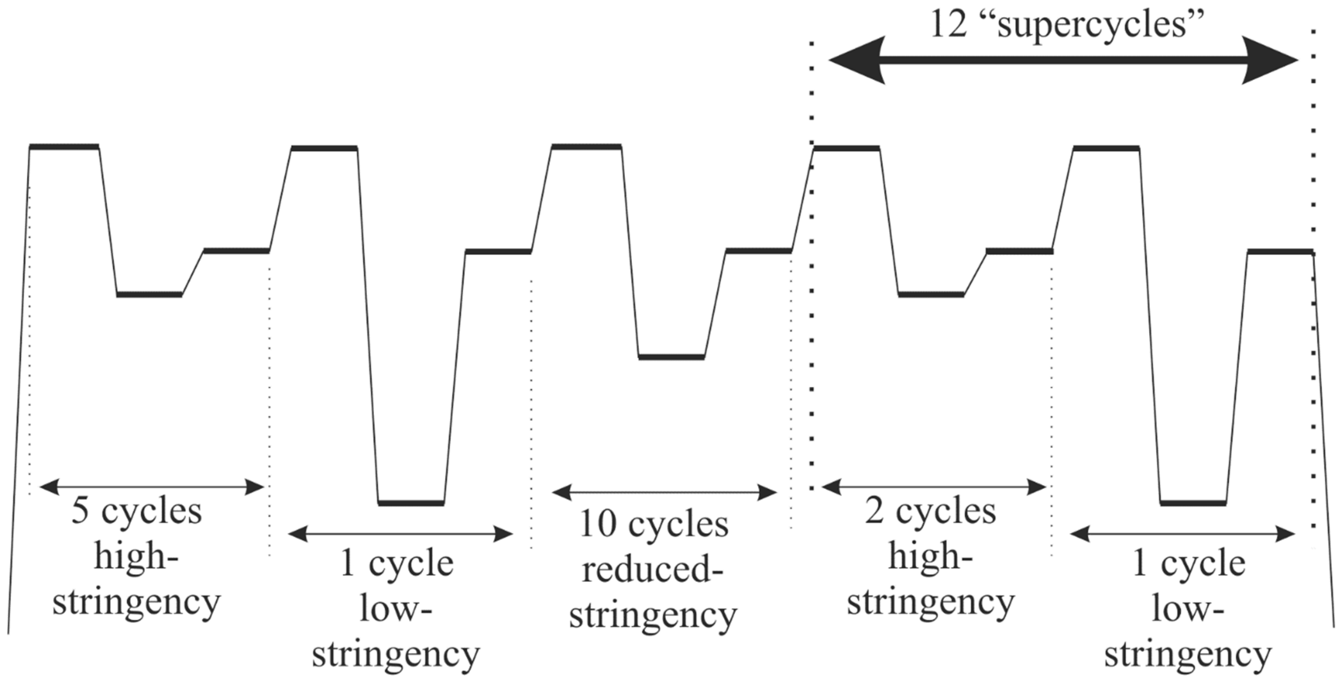

Supplemental Table S4. Published *VRN1* gene sequences for several *Poaceae* genomes were downloaded from the NCBI GenBank database:

AB630961-AB630964, AF035378, AK360697, AK376259, AY188331, AY198326, AY244509, AY280870, AY616453, AY616454, AY747597, AY747602-AY747606, DQ108934, DQ146421-DQ146423, EF591643, EF591644, EF591648, EU331672, EU331680, EU331687, EU331689, EU331690, EU331700, EU331708, EU331709, EU331723, EU331724, EU331764, EU331765, EU331770, EU331772, EU331773, EU875079, EU875080, FJ687749, FJ687750, FJ793194, GQ227988-GQ227990, GQ258851, GQ451747, GQ451757, GQ451761, GQ451770, GQ451777, GQ451779-GQ451788, GQ451791, GQ451792, GQ451797, GQ451798, GQ451802, GQ451808, GQ451809, GQ451811-GQ451814, GQ451818, GQ451820, GQ482972, GU071076, HQ130482, HQ130483, JN817430, JN817431, JN969602, JN969603, JX020755, KF939527, KF939528, KM016789, KM586653, KM586656, KM586657, KM586659, KR816809, KR816810, KT247894-KT247896, KT247898, KT750253

NCBI Accession	Information
AY616454	Aegilops tauschii bac 22j2 vrn-d1 gene, promoter and partial cds
GQ451747	Aegilops tauschii isolate asq134_d13 vernalization protein (vrn1) gene, promoter region
AB630963	Aegilops tauschii subsp. Strangulata vrn-d1 gene for mads-box protein, complete cds, strain: ku-20-9
AB630961	Aegilops tauschii subsp. Tauschii vrn-d1 gene for mads-box protein, complete cds, strain: ku-20-3
AB630962	Aegilops tauschii var. Anathera vrn-d1 gene for mads-box protein, complete cds, strain: ku-20-6
AB630964	Aegilops tauschii var. Meyeri vrn-d1 gene for mads-box protein, complete cds, strain: ku-20-10
FJ793194	Festuca arundinacea vrn1 mrna, complete cds
DQ108934	Festuca pratensis mads-box transcription factor (vrn1) gene, partial cds
FJ687750	Hordeum vulgare bio-material auspgris:aus403539 vernalization1 (vrn1) gene, complete cds
FJ687749	Hordeum vulgare bio-material auspgris:aus408347 vernalization1 (vrn1) gene, complete cds
EU331709	Hordeum vulgare subsp. Vulgare cultivar 88ab536 mads-box transcription factor bm5a (vrn-h1) gene, exon 1 and partial cds
EU331689	Hordeum vulgare subsp. Vulgare cultivar ac metcalfe mads-box transcription factor bm5a (vrn-h1) gene, exon 1 and partial cds
EU331690	Hordeum vulgare subsp. Vulgare cultivar baronesse mads-box transcription factor bm5a (vrn-h1) gene, exon 1 and partial cds
KT247898	Hordeum vulgare subsp. Vulgare cultivar br5593c3 vrn-h1 (vrn-h1) gene, complete cds
EU331724	Hordeum vulgare subsp. Vulgare cultivar canela mads-box transcription factor bm5a (vrn-h1) gene, exon 1 and partial cds
EU331764	Hordeum vulgare subsp. Vulgare cultivar dicktoo mads-box transcription factor bm5a (vrn-h1) gene, exon 1 and partial cds
EU331687	Hordeum vulgare subsp. Vulgare cultivar garnett mads-box transcription factor bm5a (vrn-h1) gene, exon 1 and partial cds
EU331700	Hordeum vulgare subsp. Vulgare cultivar harrington mads-box transcription factor bm5a (vrn-h1) gene, exon 1 and partial cds
EF591643	Hordeum vulgare subsp. Vulgare cultivar igri mads-box protein bm5a (vrn-h1) gene, complete cds
EU331708	Hordeum vulgare subsp. Vulgare cultivar kold mads-box transcription factor bm5a (vrn-h1) gene, exon 1 and partial cds
EU331765	Hordeum vulgare subsp. Vulgare cultivar kompolti korai mads-box transcription factor bm5a (vrn-h1) gene, exon 1 and partial cds
EU331772	Hordeum vulgare subsp. Vulgare cultivar luca mads-box transcription factor bm5a (vrn-h1) gene, exon 1 and partial cds
EU331773	Hordeum vulgare subsp. Vulgare cultivar merlin mads-box transcription factor bm5a (vrn-h1) gene, exon 1 and partial cds
KT247894	Hordeum vulgare subsp. Vulgare cultivar novetta vrn-h1 (vrn-h1) gene, partial cds
EU331770	Hordeum vulgare subsp. Vulgare cultivar owb-d mads-box transcription factor bm5a (vrn-h1) gene, exon 1 and partial cds
EF591644	Hordeum vulgare subsp. Vulgare cultivar panda mads-box protein bm5a (vrn-h1) gene, complete cds;
EU331672	Hordeum vulgare subsp. Vulgare cultivar robust mads-box transcription factor bm5a (vrn-h1) gene, exon 1 and partial cds
EU331723	Hordeum vulgare subsp. Vulgare cultivar shenmai3 mads-box transcription factor bm5a (vrn-h1) gene, exon 1 and partial cds
EU331680	Hordeum vulgare subsp. Vulgare cultivar steptoe mads-box transcription factor bm5a (vrn-h1) gene, exon 1 and partial cds
KT247896	Hordeum vulgare subsp. Vulgare cultivar sw alison vrn-h1 (vrn-h1) gene, partial cds
EF591648	Hordeum vulgare subsp. Vulgare cultivar xenia mads-box protein bm5a (vrn-h1) gene, complete cds
AK360697	Hordeum vulgare subsp. Vulgare mrna for predicted protein, complete cds, clone: niashv1123k08
AK376259	Hordeum vulgare subsp. Vulgare mrna for predicted protein, complete cds, clone: niashv3119f17
GU071076	Lolium multiflorum cultivar floregon vrn1 gene, partial cds
GQ227989	Lolium multiflorum cultivar gulf mads box protein 1 (vrn1) gene, complete cds
GQ227988	Lolium multiflorum cultivar tachiwase mads box protein 1 (vrn1) gene, complete cds
GQ227990	Lolium perenne cultivar manhattan mads box protein 1 (vrn1) gene, complete cds
GQ258851	Lolium perenne cultivar sr4500 mads1 (vrn1) gene, promoter region and partial cds
AY198326	Lolium perenne mads1 mrna, complete cds
JN969602	Lolium perenne retrotransposon camilla_74d14_1, partial sequence; vrn1 (lp_74d14_1) and unknown (lp_74d14_2) genes, complete cds
JN969603	Lolium perenne vrn1 (lp_7d23_1) gene, complete cds
AF035378	Lolium temulentum mads-box protein 1 (mads1) mrna, complete cds
JX020755	Mutant triticum durum isolate mutant_t4-2619 truncated vernalization protein b1 (vrn-b1) gene, complete cds
KR816809	Triticum aestivum cultivar ags 2000 (pi612956) vrn-b1 (vrn-b1) gene, complete cds

HQ130483	Triticum aestivum cultivar diamant2 vrn-b1 (vrn-b1) gene, vrn-b1-a allele, promoter region and complete cds
AY616453	Triticum aestivum cultivar langdon bac 1225d16 vrn-b1 gene, promoter and partial cds
KR816810	Triticum aestivum cultivar pioneer brand 26r61 (pi612153) vrn-b1 (vrn-b1) gene, complete cds
HQ130482	Triticum aestivum cultivar saratovskaya29 vrn-b1 (vrn-b1) gene, vrn-b1-c allele, promoter region and complete cds
AY747606	Triticum aestivum cultivar triple dirk c line vrn-d1 (vrn-d1) gene, complete cds
AY747597	Triticum aestivum cultivar triple dirk e line vrn-d1 (vrn-d1) gene, complete cds
GQ451788	Triticum aestivum isolate 100a1 vernalization protein (vrn1) gene, vrn1-a1 allele, promoter region, exon1 and partial cds
GQ451791	Triticum aestivum isolate 100b vernalization protein (vrn1) gene, vrn1-b1 allele, promoter region, exon1 and partial cds
GQ451792	Triticum aestivum isolate 100d14_27_02_09 vernalization protein (vrn1) gene, vrn1-d1 allele, promoter region, exon1 and partial cds
GQ451797	Triticum aestivum isolate 101b vernalization protein (vrn1) gene, vrn1-b1 allele, promoter region, exon1 and partial cds
GQ451798	Triticum aestivum isolate 101d_2_31_03_09 vernalization protein (vrn1) gene, vrn1-d1 allele, promoter region, exon1 and partial cds
GQ451802	Triticum aestivum isolate 102d23_27_02_09 vernalization protein (vrn1) gene, vrn1-d1 allele, promoter region, exon1 and partial cds
GQ451808	Triticum aestivum isolate 105d10_06_03_09 vernalization protein (vrn1) gene, vrn1-d1 allele, promoter region, exon1 and partial cds
GQ451809	Triticum aestivum isolate 105d8_06_03_09 vernalization protein (vrn1) gene, vrn1-d1 allele, promoter region, exon1 and partial cds
GQ451811	Triticum aestivum isolate 106b vernalization protein (vrn1) gene, vrn1-b1 allele, promoter region, exon1 and partial cds
GQ451814	Triticum aestivum isolate 113_7_3_02_09 vernalization protein (vrn1) gene, vrn1-b1 allele, promoter region, exon1 and partial cds
GQ451777	Triticum aestivum isolate 97d2_20_02_09 vernalization protein (vrn1) gene, vrn1-d1 allele, promoter region, exon1 and partial cds
GQ451779	Triticum aestivum isolate 97d7_20_02_09 vernalization protein (vrn1) gene, vrn1-d1 allele, promoter region, exon1 and partial cds
AY280870	Triticum aestivum mads-box protein tavrt-1 mrna, complete cds
KT750253	Triticum aestivum vernalization protein (vrn-b1) gene, vrn-b1dic allele, complete cds
JN817430	Triticum carthlicum genotype pi 94749 retrotransposon vrn, complete sequence; and vrn-b1 (vrn-b1) gene, complete cds
JN817431	Triticum durum cultivar lebsock vrn-b1 (vrn-b1) gene, complete cds
AY244509	Triticum monococcum cultivar g2528 vrn1 (ap1) gene, complete cds
EU875079	Triticum monococcum cultivar pi355515 mads-box transcriptional factor vrn1 (vrn1) gene, vrn1-vrn-a1b allele, complete cds
EU875080	Triticum monococcum cultivar pi573525 mads-box transcriptional factor vrn1 (vrn1) gene, vrn1-vrn-a1b allele, partial cds
AY188331	Triticum monococcum dv92 chromosome 5al bac 231a16, complete sequence
KM016789	Triticum monococcum strain pi 428170 vrn-a1 gene, exon 1, promoter region and partial cds
DQ146423	Triticum monococcum strain pi306540 vrn1 (vrn1) gene, complete cds
DQ146421	Triticum monococcum strain pi503874 vrn1 (vrn1) gene, complete cds
KM586657	Triticum monococcum subsp. Aegilopoides voucher tri 17071 vernalization protein (vrn1) gene, vrn1-a1h allele, promoter region
KM586653	Triticum monococcum voucher tri 1510 vernalization protein (vrn1) gene, vrn1-a1 allele, promoter region and partial cds
KM586656	Triticum monococcum voucher tri 28871 vernalization protein (vrn1) gene, vrn1-a1f allele, promoter region and partial cds
KF939527	Triticum spelta strain pi 428178 vrn-d1 gene, promoter region and partial cds
KF939528	Triticum spelta strain ua0300304 vrn-d1 gene, promoter region and partial cds
AY747602	Triticum turgidum cultivar langdon clone bac 1225d16 vrn-b1 (vrn-b1) gene, complete cds
GQ451820	Triticum turgidum isolate 119b vernalization protein (vrn1) gene, vrn1-b1 allele, promoter region, exon1 and partial cds
GQ451761	Triticum turgidum subsp. Dicoccon isolate 21b vernalization protein (vrn1) gene, vrn1-b1 allele, promoter region, exon1 and partial cds
GQ451757	Triticum turgidum subsp. Dicoccon isolate 9b1_20_02_09 vernalization protein (vrn1) gene, vrn1-b1 allele, promoter region, exon1
GQ451818	Triticum turgidum subsp. Turanicum isolate 118b vernalization protein (vrn1) gene, vrn1-b1 allele, promoter region, exon1
GQ451770	Triticum turgidum subsp. Turanicum isolate 34b1_17_02_09 vernalization protein (vrn1) gene, vrn1-b1 allele, promoter region
GQ482972	Triticum urartu clone 4 vrn1 (vrn1) gene, vrn1-a1 allele, exon 1 and partial cds
KM586659	Triticum urartu voucher tri 17174 vernalization protein (vrn1) gene, vrn1-a1u allele, promoter region and partial cds

Supplementary Figure S2. Multiple alignments of exon 1 sequences of the *VRN1* gene. Exon 1 is marked in red, intron 1 is marked in blue. Primers for exon 1 and their location are shown, used for the GW promoter and intron sequences.

```
           160       170       180       190       200       210       220       230       240       250
             |         |         |         |         |         |         |         |         |         |
JN969602  GGAGGGAGGCGAGATGGGGCGCGGCAAGGTGCAGCTGAAGCGGATCGAGAACAAGATCAACCGCCAGGTCACCTTCTCCAAGCGCCGCTCCGGG
JN969603  GGAGGGAGGCGAGATGGGGCGCGGCAAGGTGCAGCTCAAGCGGATCGAGAACAAGATCAACCGCCAGGTCACCTTCTCCAAGCGCCGCTCGGGG
GQ258851  GGAGGGAGGCGAGATGGGGCGCGGCAAGGTGCAGCTCAAGCGGATCGAGAACAAGATCAACCGCCAGGTCACCTTCTCCAAGCGCCGCTCGGGG
GQ227990  GGAGGGAGGCGAGATGGGGCGCGGCAAGGTGCAGCTCAAGCGGATCGAGAACAAGATCAACCGCCAGGTCACCTTCTCCAAGCGCCGCTCGGGG
AF035378  GGAGGGAGGCGAGATGGGGCGCGGCAAGGTGCAGCTCAAGCGGATCGAGAACAAGATCAACCGCCAGGTCACCTTCTCCAAGCGCCGCTCAGGC
FJ793194  GGAAGGAGGCGAGATGGGGCGCGGCAAGGTGCAGCTCAAGCGGATCGAGAACAAGATCAACCGCCAGGTCACCTTCTCCAAGCGCCGCTCCGGC
GQ227989  GGAAGGAGGCGAGATGGGGCGCGGCAAGGTGCAGCTCAAGCGGATCGAGAACAAGATCAACCGCCAGGTCACCTTCTCCAAGCGCCGCTCCGGC
DQ108934  GGAAGGAGGCGAGATGGGGCGCGGGAAGGTGCAGCTCAAGCGGATCGAGAACAAGATCAACCGCCAGGTCACCTTCTCCAAGCGCCGCTCCGGC
GU071076  GGAGGGAGGCGAGATGGGGCGCGGCAAGGTGCAGCTCAAGCGGATCGAGAACAAGATCAACCGCCAGGTCACCTTCTCCAAGCGCCGCTCCGGC
KF939528  -----GAGCGGAGATGGGGCGCGGGAAGGTGCAGCTGAAGCGGATCGAGAACAAGATCAACCGGCAGGTGACCTTCTCCAAGCGCCGCTCGGGG
AB630962  -----GAGCGGAGATGGGGCGCGGGAAGGTGCAGCTGAAGCGGATCGAGAACAAGATCAACCGGCAGGTGACCTTCTCCAAGCGCCGCTCGGGG
GQ451780  -----GAGCGGAGATGGGGCGCGGGAAGGTGCAGCTGAAGCGGATCGAGAACAAGATCAACCGGCAGGTGACCTTCTCCAGGCGCCGCTCGGGG
GQ451786  -----GAGCGGAGATGGGGCGCGGGAAGGTGCAGCTGAAGCGGATCGAGAACAAGATCAACCGGCAGGTGACCTTCTCCAAGCGCCGCTCGGGG
GQ451792  -----GAGCGGAGATGGGGCGCGGGAAGGTGCAGCTGAAGCGGATCGAGAACAAGATCAACCGGCAGGTGGCCTTCTCCAAGCGCCGCTCGGGG
GQ451813  -----GAGCGGAGATGGGGCGCGGGAAGGTGCAGCTGAAGCGGATCGAGAACAAGATCAACCGGCAGGTGACCTTCTCCAAGCGCCGCTCGGGG
GQ451798  -----GAGCGGAGATGGGGCGCGGGAAGGTGCAGCTGAAGCGGATCGAGAACAAGATCAACCGGCAGGTGACCTTCTCCAAGCGCCGCTCGGGG
GQ451788  -----GAGCGGAGATGGGGCGGGGGAAGGTGCAGCTGAAGCGGATCGAGAACAAGATCAACCGGCAGGTGACCTTCTCCAAGCGCCGCTCGGGG
GQ451808  -----GAGCGGAGATGGGGCACGGGAAGGTGCAGCTGAAGCGGATCGAGAACAAGATCAACCGGCAGGTGACCTTCTCCAAGCGCCGCTCGGGG
GQ451747  -----GAACGGAGATGGGGCGCGGGAAGGTGCAGCTGAAGCGGATCGAGAACAAGATCAACCGGCAGGTGACCTTCTCCAAGCGCCGCTCGGGG
KM586659  -----GAGCGGAGATGGGGCGCGGGAAGGTGCAGCTGAAGCGGATCGAGAACAAGATCAACCGGCAGGTGACCTTCTCCAAGCGCCGCTCGGGG
KM586657  -----GAGCGGAGATGGGGCGCGGGAAGGTGCAGCTGAAGCGGATCGAGAACAAGATCAACCGGCAGGTGACCTTCTCCAAGCGCCGCTCGGGG
KT750253  -----CAGCGGAGATGGGGCGCGGGAAGGTGCAGCTGAAGCGGATCGAGAACAAGATCAACCGGCAGGTGACCTTCTCCAAGCGCCGCTCGGGG
GQ451757  -----CAGCGGAGATGGGGCGCGGGAAGGTGCAGCTGAAGCGGATCGAGAACAAGATCAACCGGCAGGTGACCTTCTCCAAGCGCCGCTCGGGG
JN817430  -----CAGCGGAGATGGGGCGCGGGAAGGTGCAGCTGAAGCGGATCGAGAACAAGATCAACCGGCAGGTGACCTTCTCCAAGCGCCGCTCGGGG
GQ227988  GGAGGGAGGCCAGATGGGGCGCGGCAAGGTGCAGCTCAAGCGGATCGAGAACAAGATCAACCGCCAGGTCACCTTCTCCAAGCGCCGCTCGGGG
KT247898  ----------GAGATGGGGCGCGGGAAGGTGCAGCTGAAGCGGATCGAGAACAAGATCAACCGCCAGGTCACCTTCTCCAAGCGCCGCTCGGGG
AK376259  ----------GAGATGGGGCGAGGGAAGGTGCAGCTGAAGCGGATCGAGAACAAGATCAACCGCCAGGTCACCTTCTCCAAGCGCCGCTCGGGG
                                       5315 CTSAAGCGGATCGAGAACAAGATCAACC->
                                 <-TTCCACGTCGASTTCGCCTAGCTCTTG 5299

                 260       270       280       290       300       310       320       330       340       350
                   |         |         |         |         |         |         |         |         |         |
JN969602  CTGCTCAAGAAGGCGCACGAGATCTCCGTGCTCTGCGACGCCGAGGTCGGGCTCATCATCTTCTCCACCAAGGGAAAGCTCTACGAGTTCGCAACCGAC
JN969603  CTGCTCAAGAAGGCGCACGAGATCTCCGTGCTCTGCGACGCCGAGGTCGGGCTCATCATCTTCTCCACCAAGGGAAAGCTCTACGAGTTCGCAACTGAC
GQ258851  CTGCTCAAGAAGGCGCACGAGATCTCCGTGCTCTGCGACGCCGAGGTCGGGCTCATCATCTTCTCCACCAAGGGAAAGCTCTACGAGTTCGCAACTGAC
GQ227990  CTGCTCAAGAAGGCGCACGAGATCTCCGTGCTCTGCGACGCCGAGGTCGGGCTCATCATCTTCTCCACCAAGGGAAAGCTCTACGAGTTCGCAACCGAC
AF035378  CTGCTCAAGAAGGCGCACGAGATCTCCGTGCTCTGCGACGCAGAGGTCGGGCTCATCATCTTCTCCACCAAGGGAAAGCTCTACGAGTTCGCCACCGAC
FJ793194  CTGCTCAAGAAGGCGCACGAGATCTCCGTGCTCTGCGACGCCGAGGTCGGGCTCATCATCTTCTCCACCAAGGGAAAGCTCTACGAGTTCGCAACCGAC
GQ227989  CTGCTCAAGAAGGCGCACGAGATCTCCGTGCTCTGCGACGCAGAGGTCGGACTCATCATCTTCTCCACCAAGGGAAAGCTCTACGAGTTCGCAACCGAC
DQ108934  CTGCTCAAGAAGGCGCACGAGATCTCCGTGCTCTGCGACGCCGAGGTCGGGCTCATCATCTTCTCCACCAAGGGAAAGCT--------------------
GU071076  CTGCTCAAGAAGGCGCACGAGATCTCCGTGCTCTGCGACGCCGAGGTCGGGCTCATCATCTTCTCCACCAAGGGAAAGCTCTACG---------------
KF939528  CTGCTCAAGAAGGCGCACGAGATCTCCGTGCTCTGCGACGCCGAGGTCGGCCTCATCATCTTCTCCACCAAGGGAAAGCTCTACGAGTTCTCCACCGAG
AB630962  CTGCTCAAGAAGGCGCACGAGATCTCCGTGCTCTGCGACGCCGAGGTCGGCCTCATCATCTTCTCCACCAAGGGAAAGCTCTACGAGTTCTCCACCGAG
GQ451780  CTGCTCAAGAAGGCGCACGAGATCTCCGTGCTCTGCGACGCCGAGGTCGGCCTCATCATCTTCTCCACCAAGGGAAAGCTCTACGAGTTCTCCACCGAG
GQ451786  CTGCTCAAGAAGGCGCACGAGATCTCCGTGCTCTGCGACGCCGAGGTCGGCCTCATCATCTTCTCCACCAAGGGAAAGCTCTACGAGTTCTCCACCGAG
GQ451792  CTGCTCAAGAAGGCGCACGAGATCTCCGTGCTCTGCGACGCCGAGGTCGGCCTCATCATCTTCTCCACCAAGGGAAAGCTCTACGAGTTCTCCACCGAG
GQ451813  CTGCTCAAGAAGGCACACGAGATCTCCGTGCTCTGCGACGCCGAGGTCGGCCTCATCATCTTCTCCACCAAGGGAAAGCTCTACGAGTTCTCCACCGAG
GQ451798  CTGCTCAAGAAGGCGCACGAGATCTCCGTGCTCTGCGACGCCGAGGTCGGCCTCATCATCTTCTCCACCAAGGGAAAGCTCTACGAGTTCTCCACCGAG
GQ451788  CTTCTCAAGAAGGCGCACGAGATCTCCGTGCTCTGCGACGCCGAGGTCGGCCTCATCATCTTCTCCACCAAGGGAAAGCTCTACGAGTTCTCCACCGAG
GQ451808  CTGCTCAAGAAGGCGCACGAGATCTCCGTGCTCTGCGACGCCGAGGTCGGCCTCATCATCTTCTCCACCAAGGGAAAGCTCTACGAGTTCTCCACCGAG
GQ451747  CTGCTCAAGAAGGCGCACGAGATCTCCGTGCTCTGCGACGCCGAGGTCGGCCTCATCATCTTCTCCACCAAGGGAAAGCTCTACGAGTTCTCCACCGAG
KM586659  CTTCTCAAGAAGGCGCACGAGATCTCCGTGCTCTGCGACGCCGAGGTCGGCCTCATCATCTTCTCCACCAAGGGAAAGCTCTACGAGTTCTCCACCGAG
KM586657  CTTCTCAAGAAGGCGCACGAGATCTCCGTGCTCTGCGACGCCGAGGTCGGCCTCATCATCTTCTCCACCAAGGGAAAGCTCTACGAGTTCTCCACCGAG
KT750253  CTGCTCAAGAAGGCGCACGAGATCTCCGTGCTCTGCGACGCCGAGGTCGGCCTCATCATCTTCTCCACCAAGGGAAAGCTCTACGAGTTCTCCACCGAG
GQ451757  CTGCTCAAGAAGGCGCACGAGATCTCCGTGCTCTGCGACGCCGAGGTCGGCCTCATCATCTTCTCCACCAAGGGAAAGCTCTACGAGTTCTCCACCGAG
JN817430  CTGCTCAAGAAGGCGCACGAGATCTCCGTGCTCTGCGACGCCGAGGTCGGCCTCATCATCTTCTCCACCAAGGGAAAGCTCTACGAGTTCTCCACCGAG
GQ227988  CTGCTCAAGAAGGCGCACGAGATCTCCGTGCTCTGCGACGCCGAGGTCGGGCTCA---------------------------------------------
KT247898  CTGCTCAAGAAGGCGCACGAGATCTCCGTGCTCTGCGACGCCGAGGTCGGCCTCATCATCTTCTCCACCAAGGGAAAGCTCTACGAGTTCTCCACCGAG
AK376259  CTGCTCAAGAAGGCGCACGAGATCTCCGTGCTCTGCGACGCCGAGGTCGGCCTCATCATCTTCTCCACCAAGGGAAAGCTCTACGAGTTCTCAACCGAG
           <-GAGTTCTTCCGCGTGCTCTAGAGGCACG 5411           5410 CTCATCATCTTCTCCACCAAGGGAAAGCTCTACGAGTTC->
                                                            <-GAGTAGTAGAAGAGGTGGTTCCCTTTCGAGATGCTC 5412
```
